# Hemodynamic Unmasking of Atrial Septal Defect After Pulmonary Arterial Hypertension Therapy: A Pathophysiologic Perspective

**DOI:** 10.1002/ccr3.73125

**Published:** 2026-07-10

**Authors:** Nwachukwu O. Nwachukwu, Chukwuka Elendu, Chimaobi Nwevo, Davidson J. Ozoemena, Emmanuel O. Ikechukwu, Nwamaka C. Bob‐Ume, Ebiere S. Kalama

**Affiliations:** ^1^ Darlington Memorial Hospital Darlington UK; ^2^ Federal University Teaching Hospital Owerri Nigeria; ^3^ NYC Health + Hospitals/South Brooklyn Health Brooklyn New York USA; ^4^ Wayne State University Detroit Michigan USA; ^5^ Enugu State University of Science and Technology Enugu Nigeria; ^6^ All Saints University College of Medicine Kingstown Saint Vincent and the Grenadines; ^7^ Niger Delta University Amassoma Nigeria

**Keywords:** atrial septal defect, hemodynamic unmasking, multimodality cardiac imaging, pulmonary arterial hypertension, right ventricular compliance

## Abstract

Pulmonary arterial hypertension may mask underlying atrial septal defects by reducing left‐to‐right shunt flow through elevated pulmonary vascular resistance and impaired right ventricular compliance. Targeted therapy may unmask latent defects, emphasizing early multimodality imaging and sustained clinical suspicion for congenital shunts when invasive hemodynamic assessment suggests shunting despite inconclusive echocardiography.

Dear Editor,

We read with great interest the recently published case report titled “A Case of Atrial Septal Defect Unveiled by the Treatment for Pulmonary Arterial Hypertension” [1], which describes a 51‐year‐old woman with severe pulmonary arterial hypertension in whom a previously undetected secundum atrial septal defect became hemodynamically apparent following targeted pulmonary arterial hypertension therapy, allowing subsequent transcatheter closure using a treat‐and‐repair strategy.

The central message of the report—that elevated pulmonary vascular resistance (PVR) and impaired right ventricular (RV) compliance can attenuate left‐to‐right shunt flow across an ASD, thereby masking its echocardiographic detectability—is physiologically sound and consistent with established principles of interatrial shunt dynamics [2, 3]. In the described patient, severe precapillary pulmonary hypertension with a PVR of 7.4 Wood units was associated with RV pressure overload, interventricular septal flattening, and moderate tricuspid regurgitation, all of which could reasonably diminish net left‐to‐right atrial flow and obscure color Doppler detection on transthoracic echocardiography. The subsequent reduction in PVR to 2.6 Wood units following combination therapy with macitentan and tadalafil coincided with the emergence of an abundant left‐to‐right shunt, thereby “unmasking” the underlying congenital defect. This temporal relationship is persuasive and illustrates the dynamic interplay between pulmonary vascular load, ventricular compliance, and shunt magnitude (Table 1).

**TABLE 1:** Hemodynamic factors influencing detection of atrial septal defect in pulmonary arterial hypertension.

Hemodynamic phase	Pulmonary vascular resistance (PVR)	Right ventricular (RV) compliance	Atrial pressure relationship	Expected interatrial shunt flow	Echocardiographic detectability
Severe untreated PAH	Markedly elevated	Reduced due to pressure overload	Right‐sided pressures elevated; reduced left‐to‐right gradient	Attenuated or minimal left‐to‐right shunt	May be occult on transthoracic echocardiography
Early PAH treatment phase	Decreasing	Gradually improving	Rebalancing of atrial pressure gradients	Increasing left‐to‐right flow	Shunt may become detectable with Doppler or contrast imaging
After PAH therapy	Reduced	Improved RV compliance	Restoration of left‐to‐right atrial gradient	Enhanced left‐to‐right shunt flow (“hemodynamic unmasking”)	Increased likelihood of ASD visualization
Post‐ASD closure	Low or normalized	Improved remodeling	No interatrial communication	Minimal or absent residual shunt	Confirmation of closure on follow‐up imaging

Adults with severe pulmonary hypertension are often classified as having idiopathic PAH or PAH associated with systemic or thromboembolic conditions when initial noninvasive imaging fails to identify a structural cardiac lesion. As the authors correctly note, ASD‐related PAH may be clinically indistinguishable from idiopathic PAH when shunt flow is minimal due to markedly elevated right‐sided pressures. This diagnostic overlap has important consequences, as the therapeutic trajectory and long‐term prognosis of congenital heart disease–associated PAH differ substantially from those of true idiopathic disease [3, 4]. The described case reinforces the need for a high index of suspicion for occult intracardiac shunts in adults with precapillary pulmonary hypertension, particularly when right heart catheterization findings suggest shunting despite inconclusive echocardiographic evaluation.

We also find the authors' discussion of RV compliance particularly instructive. In simple secundum ASD, left‐to‐right shunting is primarily driven by the relative compliance of the ventricles and atria rather than by large pressure gradients. Conditions that reduce RV compliance—such as longstanding PAH—can therefore decrease shunt volume or even reverse shunt direction, whereas interventions that improve RV compliance may restore or increase left‐to‐right flow [3]. The reported improvement in the RV stroke volume to RV end‐diastolic pressure ratio after PAH therapy provides indirect support for improved RV diastolic properties and offers a plausible mechanistic explanation for the observed increase in shunt flow. While such indices are not routinely used in clinical practice, their inclusion enriches the physiological narrative of the case and encourages more nuanced interpretation of hemodynamic data in similar contexts.

Importantly, suspicion for an atrial septal defect should not depend exclusively on echocardiographic findings. A comprehensive diagnostic approach begins with careful history taking, physical examination, electrocardiography, and chest radiography, all of which may provide clues suggestive of an underlying left‐to‐right shunt [1, 2]. Characteristic findings such as exertional dyspnea, fixed splitting of the second heart sound, right axis deviation or incomplete right bundle branch block on electrocardiography, and cardiomegaly or prominent pulmonary vasculature on chest radiography should raise clinical suspicion and prompt further investigation. Thus, the diagnosis of ASD should not be considered missed solely because of initially nondiagnostic echocardiographic findings. Although echocardiography remains the first‐line imaging modality for suspected ASD, its sensitivity may be reduced in the presence of elevated right‐sided pressures, significant tricuspid regurgitation, or suboptimal acoustic windows [5, 6]. Accordingly, echocardiography should be viewed as one component of a broader clinical assessment, and persistent suspicion based on clinical and ancillary findings should prompt additional evaluation. As highlighted in existing guidelines and imaging position statements, adjunctive techniques such as agitated saline contrast studies, transesophageal echocardiography, cardiac computed tomography, or cardiovascular magnetic resonance imaging can substantially improve diagnostic yield, particularly when clinical suspicion for shunt disease persists despite nondiagnostic initial studies [3, 5, 7]. The authors appropriately emphasize this point in their key clinical message, and the reported case provides a vivid illustration of why multimodality imaging should be considered early in selected patients with pulmonary hypertension of unclear origin.

In recent years, accumulating evidence has supported the cautious use of targeted PAH therapy to reduce PVR before considering ASD closure in selected patients with severe pulmonary hypertension [4, 8]. By demonstrating normalization of pulmonary hemodynamics and favorable short‐term outcomes following staged medical therapy and device closure, the authors contribute additional real‐world support for this approach. At the same time, the case also serves as a reminder that such strategies require careful patient selection, meticulous hemodynamic reassessment, and close long‐term follow‐up, particularly in light of guideline recommendations that advise against defect closure in patients with persistently elevated PVR despite adequate medical therapy [3, 9].

We would, however, suggest that the phenomenon described in this report may be less novel than it initially appears, although it remains underrecognized in routine clinical practice. Prior case reports and small series have documented the detection of previously unrecognized ASDs following treatment of pulmonary hypertension from various etiologies, including portopulmonary hypertension [10]. These observations collectively suggest that the “unmasking” of shunt lesions after reduction of pulmonary vascular load is a reproducible physiological event rather than an isolated curiosity. Framing this case within this emerging body of literature may help clinicians appreciate that improvement in pulmonary hemodynamics can reveal, rather than create, intracardiac shunts, and that the absence of echocardiographic evidence at baseline does not definitively exclude congenital heart disease in this population.

In conclusion, atrial septal defects may remain clinically and echocardiographically occult in the setting of severe pulmonary arterial hypertension and become hemodynamically apparent following reduction of pulmonary vascular resistance. The emergence of left‐to‐right shunting after targeted PAH therapy underscores the dynamic nature of shunt physiology. Maintaining a high index of suspicion and incorporating multimodality imaging when clinical or hemodynamic findings suggest intracardiac shunting may improve diagnostic accuracy and guide appropriate management.

## Author Contributions

**Nwachukwu O. Nwachukwu:** visualization, writing – review and editing. **Chukwuka Elendu:** conceptualization, data curation, formal analysis, investigation, methodology, project administration, supervision, visualization, writing – original draft, writing – review and editing. **Chimaobi Nwevo:** validation, writing – review and editing. **Davidson J. Ozoemena:** visualization, writing – review and editing. **Emmanuel O. Ikechukwu:** validation, visualization. **Nwamaka C. Bob‐Ume:** validation, writing – review and editing. **Ebiere S. Kalama:** validation, visualization.

## Funding

The authors have nothing to report.

## Ethics Statement

Ethical approval was not required, as this letter does not involve new patient data, human subject research, or original clinical investigation.

## Consent

Written informed consent for publication was obtained by the authors of the original report. This letter does not include new patient data.

## Conflicts of Interest

The authors declare no conflicts of interest.

## Data Availability

No datasets were generated or analyzed for this letter.
